# Necrosis, apoptosis, necroptosis, three modes of action of dopaminergic neuron neurotoxins

**DOI:** 10.1371/journal.pone.0215277

**Published:** 2019-04-25

**Authors:** Noëlle Callizot, Maud Combes, Alexandre Henriques, Philippe Poindron

**Affiliations:** Department of Pharmacology, Neuro-Sys SAS, Gardanne, France; University of Nebraska-Lincoln, UNITED STATES

## Abstract

Most of the Parkinson’s disease (PD) cases are sporadic, although several genes are directly related to PD. Several pathways are central in PD pathogenesis: protein aggregation linked to proteasomal impairments, mitochondrial dysfunctions and impairment in dopamine (DA) release. Here we studied the close crossing of mitochondrial dysfunction and aggregation of α-synuclein (α-syn) and in the extension in the dopaminergic neuronal death. Here, using rat primary cultures of mesencephalic neurons, we induced the mitochondrial impairments using “DA-toxins” (MPP^+^, 6OHDA, rotenone). We showed that the DA-Toxins induced dopaminergic cell death through different pathways: caspase-dependent cell death for 6OHDA; MPP^+^ stimulated caspase-independent cell death, and rotenone activated both pathways. In addition, a decrease in energy production and/or a development of oxidative stress were observed and were linked to α-syn aggregation with generation of Lewy body-like inclusions (found inside and outside the dopaminergic neurons). We demonstrated that any of induced mitochondrial disturbances and processes of death led to α-syn protein aggregation and finally to cell death. Our study depicts the cell death mechanisms taking place in *in vitro* models of Parkinson’s disease and how mitochondrial dysfunctions is at the cross road of the pathologies of this disease.

## Introduction

Parkinson’s disease (PD) is a common neurodegenerative movement disorder that affects around 1% of the population over the age of 70[1]. It is the second most common neurodegenerative disease after Alzheimer’s disease. The patients suffering from PD display symptoms of motor instabilities with resting tremor as the first symptom in 70% of the cases. Other clinical symptoms are rigidity, bradykinesia and postural instability, and often include cognitive impairment, depression and sleep disorders[2].

The etiology of PD remains unknown. Genetic, environmental risk factors and their interaction play a major role in PD. Although 90% of PD cases are sporadic, several genes exist which are directly related to inherited cases of PD. Mutations in the alpha-synuclein (α-syn) gene and the leucine rich repeat kinase LRRK2 (PARK8 gene) cause autosomal dominant PD, while mutations in Parkin (PARK2 gene), PINK1 (PARK6 gene) or DJ-1 (PARK7 gene) cause autosomal recessive PD [3–5]. Increased risk factor for PD is also found in carriers of heterozygous mutations in GBA1[6]. In parallel, it has been well established that intoxication with dopaminergic neuron-specific toxins (DA-toxins), including 1–methyl-4-phenyl-1,2,3,6 tetrahydropyridine (MPTP), 6-hydroxydopamine (6OHDA) or rotenone, cause parkinsonism in humans[7]. These toxins are used by scientists to experimentally mimic PD in animals.

Although the pathological mechanisms leading to *substantia nigra* (SN) degeneration might be multiple, several pathways are probably central: protein aggregation linked to proteasomal impairments, mitochondrial dysfunction, and impairment in dopamine (DA) release. All these pathways could converge and generate reactive oxygen species (ROS) and oxidative stress resulting in cell death[8].

In this study, we decided to work on primary cultures of mesencephalic neurons. As we have already observed and mentioned [9], these cultures are composed of 70% neurons (mainly GABAergic), 8% of which are dopaminergic neurons. For this reason, in order to specifically address these dopaminergic neurons, we could not apply global investigation techniques (such as WB or qPCR) involving whole cell pellets. We have deliberately chosen immunohistochemistry, allowing us to specifically investigate targeted neurons with double staining, by applying stringent conditions for the numbering of labelled cells.

We showed that DA-toxins induced a decrease in energy production, oxidative stress linked to α-syn aggregation and generation of Lewy body (LB)-like inclusions, and finally dopaminergic neuronal death through different cell death pathways. We also showed that whatever the mechanisms of cell death observed (apoptosis and/or necrosis or necroptosis), α-synuclein (α-syn) production and aggregation always occurred. Additionally, α-syn aggregates is well known to induce some mitochondrial dysfunctions. Thus, we hypothesized that whatever the initial causes of TH-expressing neuron death, production of extracellular aggregated α-syn triggered subsequent mitochondrial impairments. Therefore, mitochondria and α-syn could be linked in an endless vicious circle leading to the inevitable death of neurons.

## Materials and methods

### Primary culture of mesencephalic neurons

The collection of embryos was carried out in accordance with the National Institutes of Health Guide for the Care and Use of Laboratory Animals and followed current European Union regulations (Directive 2010/63/EU) and was supervised and approved by the local direction of the veterinary services of the Bouches-du-Rhône (agreement number A1301310).

Pregnant female rats of 15 days gestation (Rats Wistar; Janvier Labs France) were killed using a deep anesthesia with CO_2_ chamber followed by cervical dislocation.

Rat dopaminergic neurons were prepared and cultured as previously described by Visanji and colleagues [9]. Briefly, the midbrains obtained from 15-day old rat embryos (Wistar, Janvier Labs, St. Berthevin, France) were dissected under a stereo zoom binocular microscope. The embryonic midbrains were removed and placed in ice-cold L15 medium of Leibovitz (L15, Pan Biotech, Aidenbach, Germany) containing 2% of penicillin (10,000 U/mL) and streptomycin (10 mg/mL) solution (PS, Pan Biotech) and 1% of bovine serum albumin (BSA, Pan Biotech). The ventral portion of the mesencephalic flexure, a region of the developing brain rich in dopaminergic neurons, was used for the cell preparations.

The midbrains were dissociated by trypsinization for 20 min at 37°C (Trypsin 0.05% EDTA 0.02%, Pan Biotech). The reaction was stopped by the addition of Dulbecco’s modified Eagle’s medium (DMEM, Pan Biotech) containing 0.1 mg/mL of DNase I grade II (Pan Biotech) and 10% of fetal calf serum (FCS, Invitrogen, Cergy Pontoise, France). Cells were then mechanically dissociated by 3 passages through a 10 mL pipette. They were then centrifuged at 180 x *g* for 10 min at +4°C on a layer of BSA (3.5%) in L15. The supernatant was discarded and the cell pellets were re-suspended in a defined culture medium consisting of Neurobasal medium (Invitrogen) added with 2% of B27 complement (Invitrogen), 2 mmol/L of L-glutamine (Pan Biotech), 2% of PS solution, 10 ng/ mL of Brain-derived neurotrophic factor (BDNF, Pan Biotech) and 1 ng/ mL of glial-derived neurotrophic factor (Pan Biotech). Viable cells were counted in a Neubauer cytometer using the trypan blue exclusion test. The cells were seeded at a density of 40,000 cells/well in 96 well-plates pre-coated with poly-L-lysine (Corning Biocoat, Le Pont de Claix, France) or 225,000 cells/well in 24 well-plates (Greiner, Courtaboeuf, France) coated with poly-L-lysine ([10 μg/mL, for 2 h], Sigma Aldrich) and maintained in an air (95%)-CO_2_ (5%) humidified incubator, at 37°C. Half of the medium was replaced every 2 days with fresh medium. The mesencephalic cell cultures were treated after 6 days of culture. The dopaminergic neurons were identified by immunostaining of TH. As mentioned by Visanji and colleagues [9], in our conditions, TH-expressing neurons represented 6% to 8% of neuronal population, although under these conditions, a residual population of microglial cells remain, accounting for ~1% of all cells.

### Treatment of primary culture

The medium was removed and replaced with fresh medium containing or not, either 1-methyl-4-phenyl-pyrididium (MPP^+^, at 2, 4, 8 and 16 μmol/L), a metabolite of MPTP, 6OHDA (at 5, 10, 20 and 30 μmol/L) or rotenone (0.1, 1, 10 and 20 nmol/L) (all from Sigma Aldrich) at different concentrations, for different times of incubation (6 wells per condition). Here, these three toxins were used to mimic in cell cultures *in vitro* cytopathic effects observed in the brains of PD-suffering patients. They were dissolved in the defined culture media mentioned above. Involvement of poly-ADP-ribose polymerase (PARP-1), a partner involved in necroptosis, was assessed using AG14361 (Selleckchem, Houston, Texas, USA), a specific inhibitor of PARP-1 (1 μmol/L, added to the cultures 1 h before DA-toxins).

### Immunostaining

#### General procedure

After intoxication, the cells were washed with phosphate-buffered saline (PBS; Pan Biotech) and fixed with a solution of 4% paraformaldehyde (PFA; Sigma Aldrich) in PBS, pH 7.3, for 20 min at room temperature (RT). They were washed twice again in PBS, and were then permeabilized and the non-specific sites blocked using a solution of PBS containing 0.1% of saponin (Sigma Aldrich) and 1% of FCS, for 15 min at RT. All primary antibody (Ab) incubations were performed in PBS containing 1% of FCS, 0.1 mg/mL of saponin, for 2 h, at RT. The cells were then washed with PBS containing 1% of FCS, 0.1% of saponin, and incubated with the appropriate secondary Ab (goat anti-mouse, labeled with Alexa 488 or goat anti-rabbit, labeled with Alexa 568, Invitrogen), used at 5 μg/mL in PBS containing 1% of FCS and 0.1% of saponin, for 1 hour at RT. The different Ab, and detailed procedures used throughout this study are listed in S1 Table.

#### Labeling TH-expressing neurons

TH-expressing (TH+) neurons, here, stand for “TH-immunoreactive neurons”. TH is involved in the conversion of phenylalanine into DA. Survival of TH-expressing neurons was expressed in terms of neuronal cell bodies labelled for this enzyme and was evaluated 24 hours and 48 hours after toxin application, unless otherwise mentioned.

#### Double labelling

Colocalization of markers susceptible to be linked to cytotoxic effects of neurotoxins was detected by labeling cultures, already marked for TH, for one of the following molecules: (a) Cytochrome-C (CytC) was detected using an anti-CytC rabbit polyclonal Ab, in order to evaluate the effects of neurotoxins on the mitochondrial death pathway, (b) Caspase 3 was detected with anti-activated caspase 3 rabbit polyclonal Ab to evaluate the extent of apoptosis, (c) Apoptosis Inducing Factor (AIF) was detected using an anti-AIF rabbit polyclonal Ab, (d) LC3B was detected with a rabbit polyclonal anti-LC3B (e) α-synuclein was detected with a rabbit polyclonal anti-αsyn. For the quantitative studies of marker colocalization, only TH-expressing neurons were taken into consideration.

#### Evaluation of ROS production

ROS production was evaluated by imaging, using the CellROX reagent (Thermo Fisher Scientific branch, Illkirch, France) following the procedure described by the manufacturer. CellROX reagent contains a probe that become fluorescence in contact of reactive oxygen species.

#### Quantitative analyses of immunolabeled cultures with cell analyzer

The immunolabeled cultures were automatically examined with ImageXpress (Molecular Devices, San Jose, CA, USA) equipped with a LED at 10x magnification. For the detection of fluorescence, the cell analyzer took into consideration cells presenting a signal equal to/ or greater than/ a threshold automatically fixed by observation of cultures treated only with secondary antibodies. This threshold is very low, since secondary antibodies did not label the cells. All wells were observed exactly under the same conditions, so as the objects detected by the cell analyzer belonged to the same class, rendering possible statistical comparisons.

For each condition (6 culture wells), 20 automatically selected fields per well (representing 80% of the total surface of the well) were automatically analyzed using MetaXpress software (Molecular Devices) to determine the total number of TH-expressing neuronal cell bodies, the number of neuronal cell bodies co-expressing TH along with Cyt-C or caspase 3.

Area of cytoplasmic of α-synuclein or RIPK3 was detected in TH expressing neurons. In addition, the area of AIF1 in the nucleus and the area of the dot-like LC3 signal in the cytoplasm of TH-expressing neurons were determined. The results were expressed in terms of percentage of neurons displaying the character analyzed, as compared to control conditions. Preliminary experiments have shown that secondary Abs never stained the cultured cells. Therefore, the negative control, that is the threshold of negativity, was determined by examination of cultures after immunostaining.

### Global evaluation of necrosis and apoptosis

Induction of necrosis and apoptosis were evaluated with ApoTox Glo assay kit (Promega, Madison, WI, USA) according to the manufacturer’s recommendations. Briefly, the degradation of a cell impermeant substrate, by proteases released from dead cells, was used as proxy for necrosis levels. Similarly, apoptosis level was assessed with the presence of a aminoluciferin, produced by the degradation of its precursor by caspase-3/7 activity.

### ATP production

After neurotoxic treatment, ATP levels were quantified with Cell titer Glo assay kit (Promega) according to the manufacturer’s recommendations. The assay is based on the recording of luminescence generated by an ATP-dependent luciferase. ATP level were determined for the whole culture and was normalized to cell viability.

### Hematein-eosin staining

Lewi bodies were observed using the hematein-eosin staining. After fixation, the cells were incubated 3 min at RT with hematein (Sigma Aldrich). They were washed twice in water and then washed twice again in ultra-pure water. The cells were incubated 5 min at room temperature with eosin (Sigma-Aldrich). They were washed once in water and they were then washed three times again in ultra-pure water.

### Statistical analyses

In order to express the level of cytopathic effects, data were expressed in percentage of control conditions (no intoxication or no α-syn = 100%). All values were expressed as mean +/- SEM (standard error of the mean) of 6 wells. Statistical analyses were done for the different conditions studied, using ANOVA followed by Dunnett’s test when allowed, using GraphPad Prism, version 7,04 (GraphPad software Inc, La Jolla, CA, USA).

## Results

### Effects of the DA-toxins on survival of TH-expressing neurons

In a preliminary study, effects of MPP^+^, 6OHDA and rotenone, on survival of TH-expressing neurons were assessed after 24 hours and 48 hours of intoxication. MPP^+^ mainly acts as a mitochondrial complex I inhibitor; it has been widely used to induce PD-like symptoms in animals. The neuron toxin 6OHDA has the longest history of use as a PD model and has been commonly used to degenerate central catecholaminergic projections, including the nigrostriatal system [10]. Rotenone, a natural pesticide, was proven to provoke death of dopaminergic neurons. After being administered to animals, these toxins induce the production of LB–like inclusions in their brains. LB mainly consists of aggregated α-syn. Here, these three toxins were used to mimic in cell cultures *in vitro* cytopathic effects observed in the brains of PD-suffering patients.

Whichever type of death they underwent, it was considered that a decrease in number of soma and disappearance of neurite network of TH-expressing neurons was a relevant expression of their death (see recommendations of [11]).

**Fig 1:**
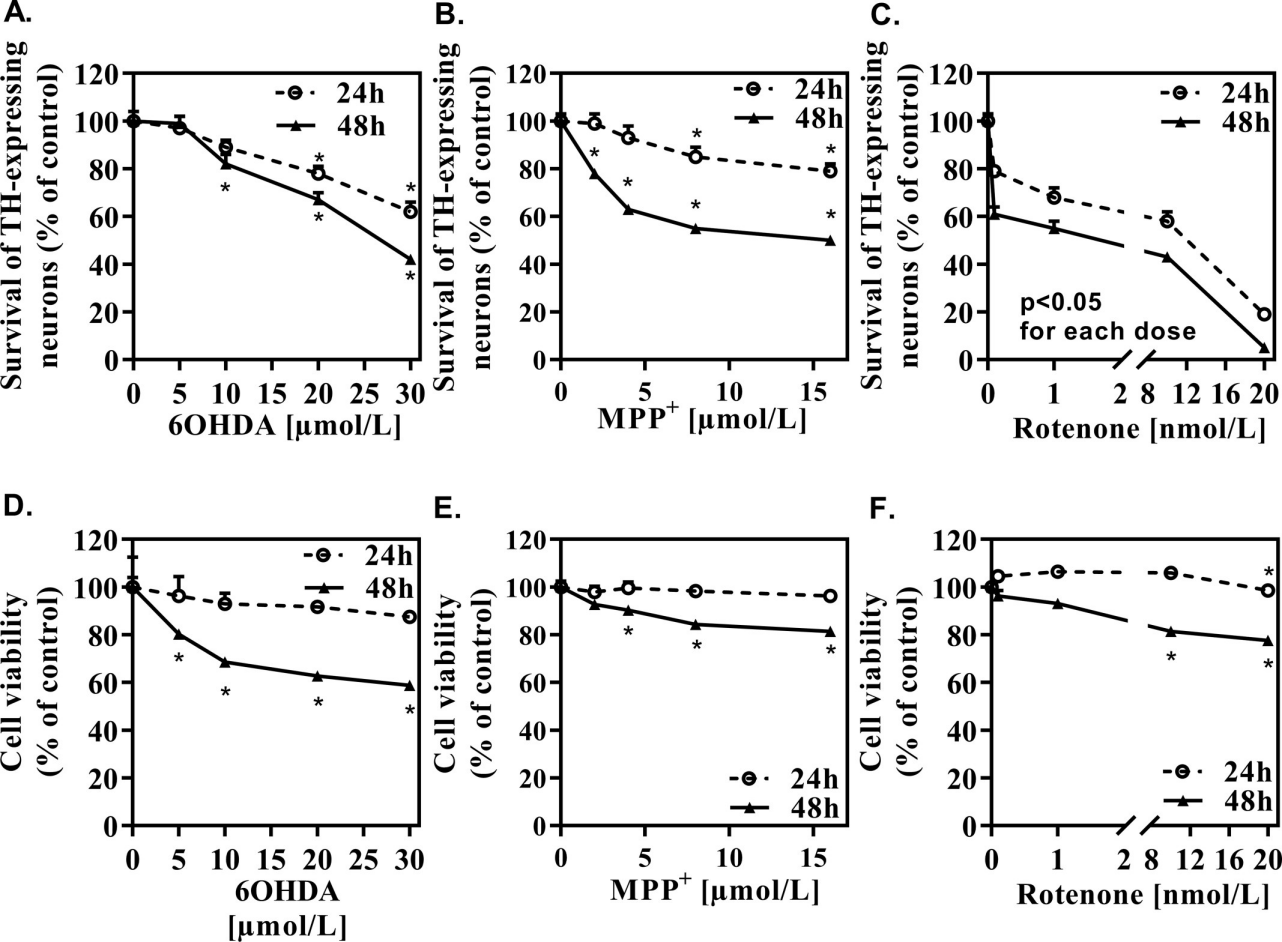
Effect of the different doses of DA-toxins on TH-expressing neurons and cell viability. Survival of TH expressing neurons after application of 6OHDA (A), MPP^+^ (B) and Rotenone (C) was studied after 24 hours and 48 hours. 100% = 61.2 +/- 1. Cell viability in a total mesencephalic culture after application of 6OHDA (D), MPP^+^ (E) and rotenone (F). All values were expressed as mean +/- SEM; n = 6/group; *, p<0,05 with One-way ANOVA followed by Dunnett’s test.

As shown in Fig 1, 6OHDA (**Fig 1A)** showed a lethal effect which slightly and progressively increased between 10 μmol/L and 30 μmol/L. No major differences between the results observed at 24 hours and 48 hours were observed except for the highest dose (30 μmol/L).

By contrast, the maximal toxic effect of MPP^+^ evolved with time (**Fig 1B**); after 24 hours of treatment, only 20% of TH-expressing neurons were killed at the dose of 16 μmol/L (the maximal concentration tested) whereas after 48 hours of treatment, this proportion reached 40%.

Rotenone (**Fig 1C**), the most toxic component, already caused 40% of TH-expressing neurons to disappear at the lowest dose of 0.1 nmol/L and almost 100% at 20 nmol/L, the maximal dose tested, after 48 hours of intoxication. Here again, no major difference was observed between 24 hours and 48 hours. The lethal effect of rotenone sharply increased beyond 10 nmol/L.

In addition to the survival of TH expressing neurons, we have assessed total cell viability, in presence of the three DA-toxins (**Fig 1D–1F)**. After 24 hours of intoxication, no significant decrease in cell viability was observed. Lower viability was however observed after 48 hours of intoxication with all DA-toxins, but this decrease was modest when compared to the toxicity observed on TH-expressing neurons.

Altogether, although the three toxins are known to inhibit complex I of the mitochondrial respiratory chain, their molecular targets in the complex or in the neuron are probably different and cannot be limited to their sole mitochondrial effects. These observations prompted us to determine which kind of death, apoptosis, necrosis or other mode they induced.

### Evaluation of necrosis and apoptosis in a primary culture of mesencephalic cells, upon intoxication with DA-toxins

**Fig 2:**
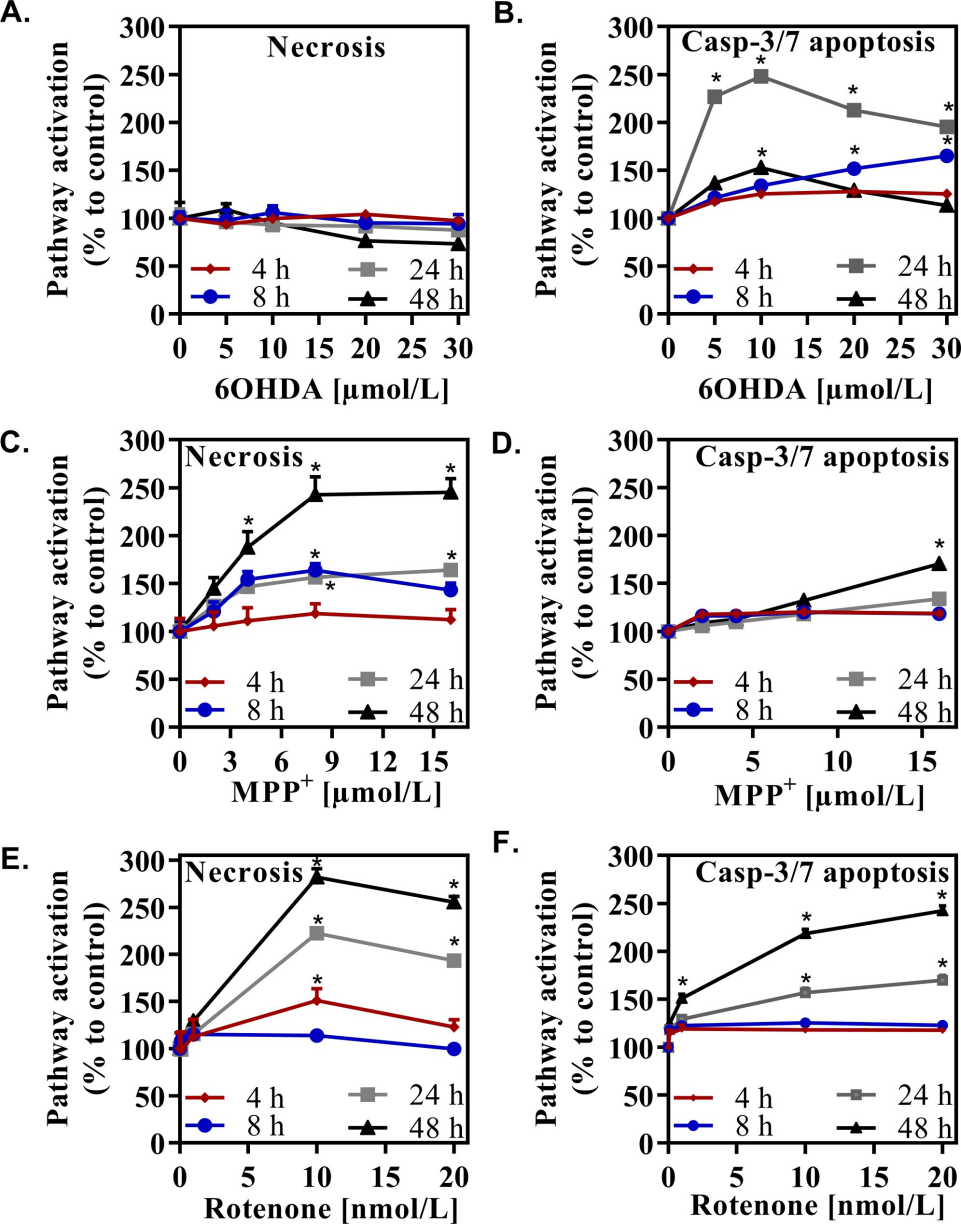
Effect of the different doses of DA-toxins on necrosis and apoptosis. Cell deaths in a total mesencephalic culture induced by 6OHDA (A, B), MPP^+^ (C, D) or rotenone (E, F) is shown. Value of 1 corresponds to the mean intensity of luminescence or fluorescence in the vehicle condition. All values were expressed as mean +/- SEM; n = 6/group; *, p<0,05 with One-way ANOVA followed by Dunnett’s test.

We have first assessed the regulation of necrosis (assessed by loss of cell membrane integrity) and apoptosis (assessed by caspase-3/7 activity) by the three toxins. As shown in **Fig 2A and 2B**, 6OHDA killed mesencephalic cells by inducing apoptosis and had no pro-necrotizing effect. The apoptotic effect was dose-dependent and reached its maximum (about 2,5 folds) after a 24-hour intoxication. Beyond this time, cell bodies of dead cells probably fragmented and disappeared from the culture. Hence, the apparently paradoxical results observed after 48 hours of contact with 6OHDA, where the percentage of apoptotic cells fell to about 1.6-fold. By contrast, MPP^+^ killed mesencephalic cells by inducing a cell death independent on apoptosis and partially dependent on necrosis.

This necrotic effect plateaued (2.4 folds, 48 hours of intoxication) at the concentration of 8 μmol/L. It sharply increased after 24 hours of intoxication (**Fig 2C**). By contrast, MPP^+^ did not massively trigger apoptosis. The maximal induction of apoptosis (1.7 folds when compared to vehicle) was observed after 48 hours of intoxication with the highest dose of MPP^+^ (**Fig 2D**). Other doses of MPP^+^ triggered a limited induction of apoptosis, by 1.2 to 1.3 folds. Finally, rotenone exerted both apoptotic and necrotizing effects (expressed as indicated above) in a dose-dependent manner. Rotenone-induced necrosis was massive (2.9 folds, 48 hours of intoxication, 10 nmol/L) and began rather early (8 hours) (**Fig 2E**). The massive necrotic effect explained why at 20 nmol/L, a lesser percentage of TH-expressing neurons was detected. A large proportion of necrotic neurons had already disappeared from the culture at this late stage. Apoptotic effects began to be significant after 24 hours of intoxication and reached its maximum (about 2.4 folds; 20 nmol/L) after 48 hours (**Fig 2F**).

Next, we sought to further delineate the regulation of programmed cell death and sought to determine whether TH-expressing neurons showed an increased level of caspase3 and CytC after 24 hours of 6OHDA (20 μmol/L), MPP^+^ (4 μmol/L) or rotenone (10 nmol/L) application. These doses and time point were chosen as they were able to engage cell death mechanisms without dramatic cell death. The role of CytC in apoptosis is well established (for review see [12]). Its translocation, from mitochondria to cytoplasm, activates caspases involved in apoptosis.

**Fig 3:**
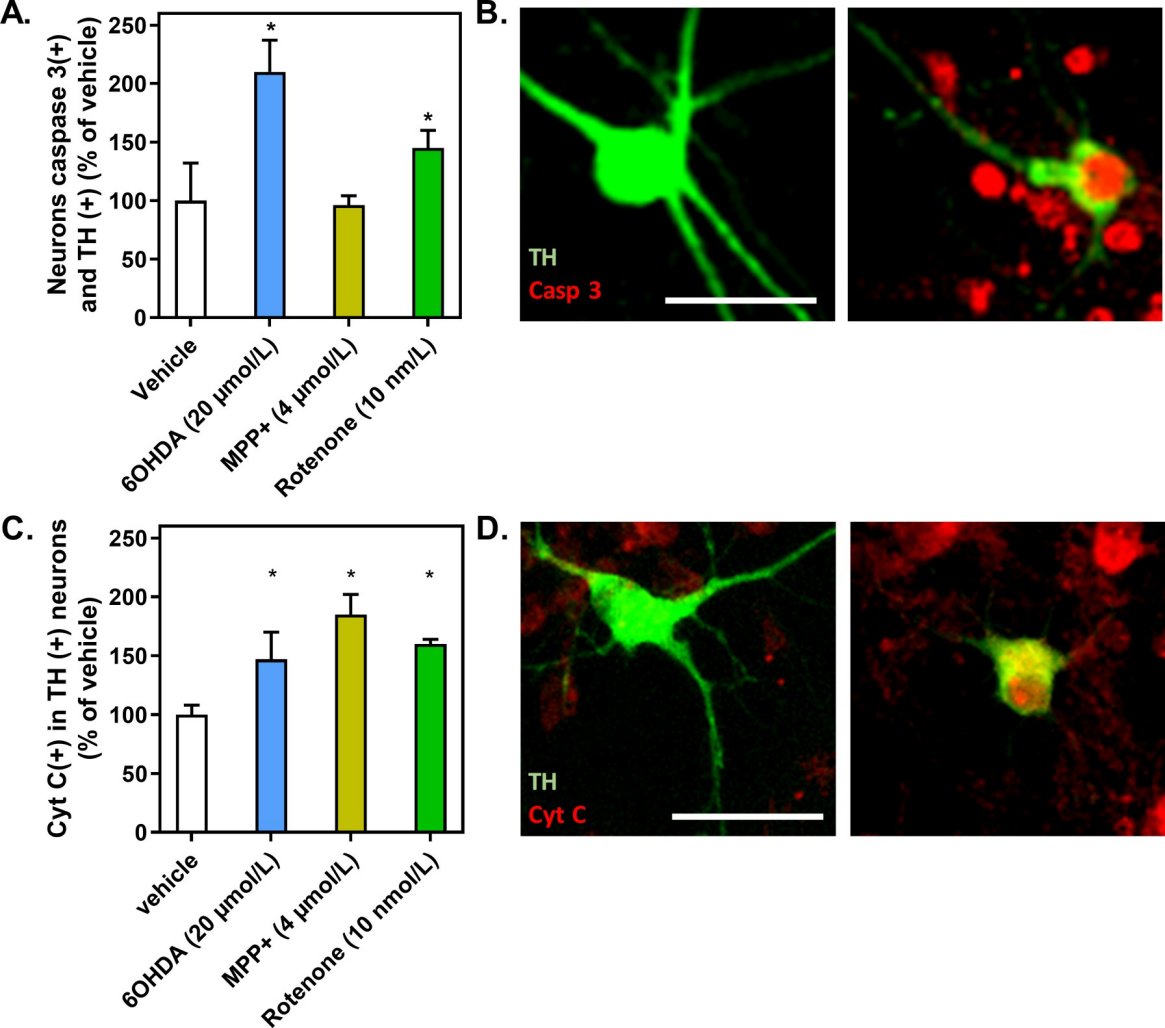
Caspase 3 and cytochrome C in dopaminergic neurons upon DA-toxins application. (A) Quantification of mesencephalic neurons positive for TH and caspase 3 staining. (B) Representative pictures of TH neurons, negative (left panel) and positive (right panel) for cleaved caspase 3. (C) Quantification of mesencephalic neurons positive for TH and cytochrome C staining. (D) Representative pictures of TH neurons, negative (left panel) and positive (right panel) for cytochrome C. Value of 1 is equivalent to 8 +/- 0,4 neurons (A) or to 5 +/- 0,4 neurons (C). All values were expressed as mean +/- SEM; n = 6/group; *, p<0,05 with One-way ANOVA followed by Dunnett’s test.

The number of caspase3-positive TH- expressing neurons was significantly increased upon 6OHDA (1.1 folds0%) or rotenone (1.45 folds) application, while MPP^+^ had no effect when compared to the control (**Fig 3A and 3B**). These results are highly similar to those obtained by measuring caspase-3/7 activity described above. The three toxins induced an increase in CytC levels, 24 hours after application (**Fig 3C and 3D**). This effect was an early event after MPP^+^ and rotenone application, as a release of CytC was observed after 8 hours (S1 Fig).

Altogether, these results suggest 6OHDA mainly elicits caspase-dependent apoptosis while MPP^+^ markedly triggers necrosis. Regarding rotenone, both investigated pathways were strongly stimulated.

### Effects of the DA-toxins on non-apoptotic cell death in TH-expressing neurons

Increasing evidence, from pre-clinical and clinical studies, suggests a role for caspase-independent cell death pathways in the loss of dopaminergic neurons in Parkinson’s disease [13,14]. Here, we sought to investigate the regulation of necroptosis and parthanatos, two caspase-independent cell death pathways, in TH neurons after injury with 6OHDA, MPP^+^ or rotenone.

The activation of necroptosis will result in the formation of the complex of proteins, the necrosome, composed by the proteins RIPK1, RIPK3, FADD and inactive caspase 8. The necrosome complex will in turn promote the phosphorylation of MLKL, trough the action of RIPK3, and its addressing to the plasma membrane to promote cell membrane permeability and cell death. Plasma membrane disruption, and cell death, occurs rapidly after the phosphorylation of MLKL by RIPK3 [15].

**Fig 4:**
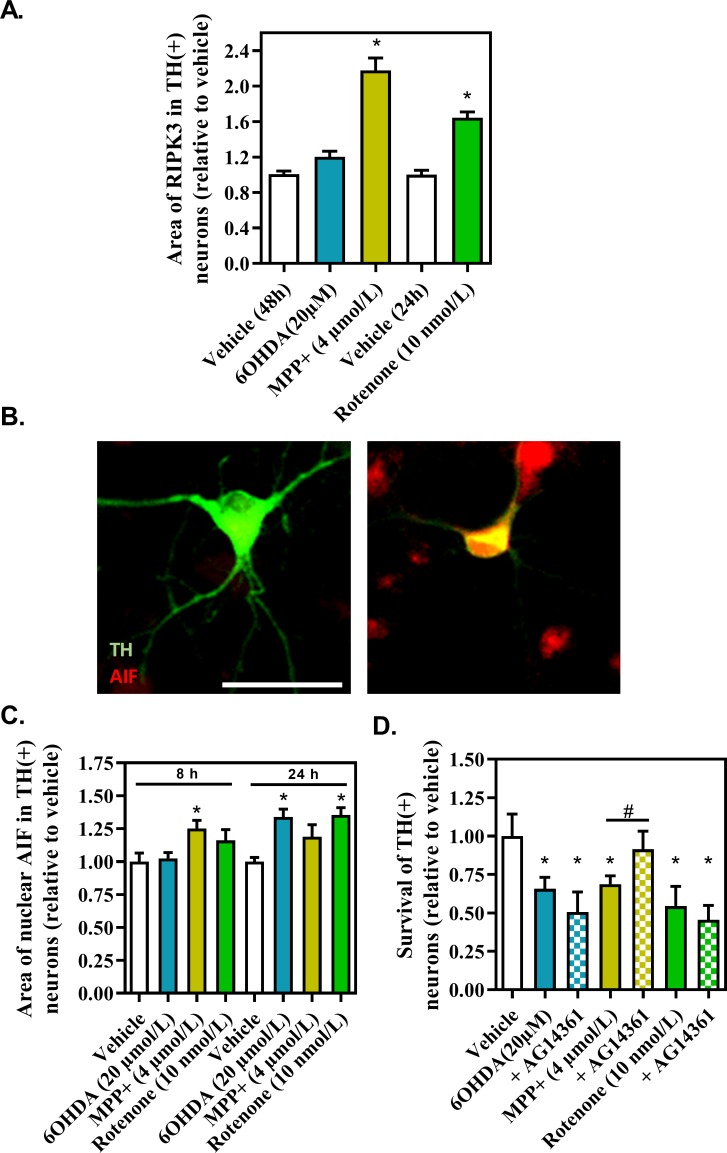
Effect of the DA-toxins on necroptosis pathway. (A and B) AIF staining in TH expressing neurons and representative pictures. (B). Survival of TH expressing neurons upon intoxication and treatment with AG14361 (1μmol/L), an inhibitor of PARP1. The value of 1 correspond to 9 +/- 0.6 neurons (AIF and TH neurons) or 15 +/- 0.6 neurons (TH neurons). All values were expressed as mean +/- SEM; n = 6/group; *, p<0,05 with One-way ANOVA followed by Dunnett’s test.

For this reason, the regulation of RIPK3, a specific effector of necroptosis, was investigated by immunohistochemistry after 48-hours of exposure to 6OHDA and MPP^+^, and after 24 hours of exposure to rotenone, as previously done by Liu and colleagues (Fig 4A) [16].

At these respective timepoints, significant, but not massive, toxicity was observed in presence of the toxins (i.e. close to 60%).

A large and significant increase in RIPK3 cytoplasmic area was observed after an application of 48 hours of MPP^+^, suggesting that the necroptosis pathway was strongly activated. In addition, a modest but significant increase in RIPK3 signal was also observed after a 24-hour application of rotenone, probably linked to massive ROS production. After the 48-hour application of 6OHDA, no increase of RIPK3 signal was observed in TH neurons.

Parthanatos is usually triggered by DNA damage due to alkylation or oxidative stress, which result in the release of mitochondrial AIF by PARP1 and its translocation into the nucleus, where AIF acts as the key actuators of this phenomenon and DNA fragmentation. AIF plays the role of a true “Janus” molecule, since it is endowed with red-ox activity in the mitochondria and with an apoptotic-like activity in the nucleus [17].

The presence of AIF in the nucleus of TH-expressing neurons was investigated after application of the DA-toxins. The translocation of AIF into the nucleus was observed in TH-expressing neurons cultured for 8hours with MPP^+^. A similar translocation was observed after an application of 6OHDA and rotenone but only after 24 hours, suggesting that parthanatos is a source of toxicity after an intoxication with these toxins (**Fig 4B and 4C**).

PARP-1 belongs to a family of nuclear enzymes, modulating DNA repair, transcriptional regulation, chromatin modification and genomic stability through polyADP-ribosylation [18]. PARP-1 over-activation leads to progressive ATP-depletion, preventing energy-dependent apoptosis [19]. PARP-1 activation promotes the cleavage of AIF and its translocation into the nucleus.

To determine whether the AIF pathway significantly contributes to the loss of TH-expressing neurons, we have investigated the toxicity of the three DA-toxins in presence of AG14361 an inhibitor specific of PARP-1 (**Fig 4D**).

AG14361 abolished the injury induced by MPP^+^ (after a 48-hour application). In contrast, it did not protect against toxic effects of 6OHDA or rotenone.

These results provide the experimental evidence that caspase-independent cell death pathway were involved in the loss of nigral dopaminergic neurons caused by administration of MPP^+^, which is in agreement with the literature [13].

### Mitochondrial pathology in presence of the DA-toxins

Mitochondrial pathology will shortly translate into abnormal level of ATP and higher reactive oxygen species (ROS) production. As these events are early, we have extended the duration of the study, to include predominantly alive cells while under the toxic action of the drugs.

**Fig 5:**
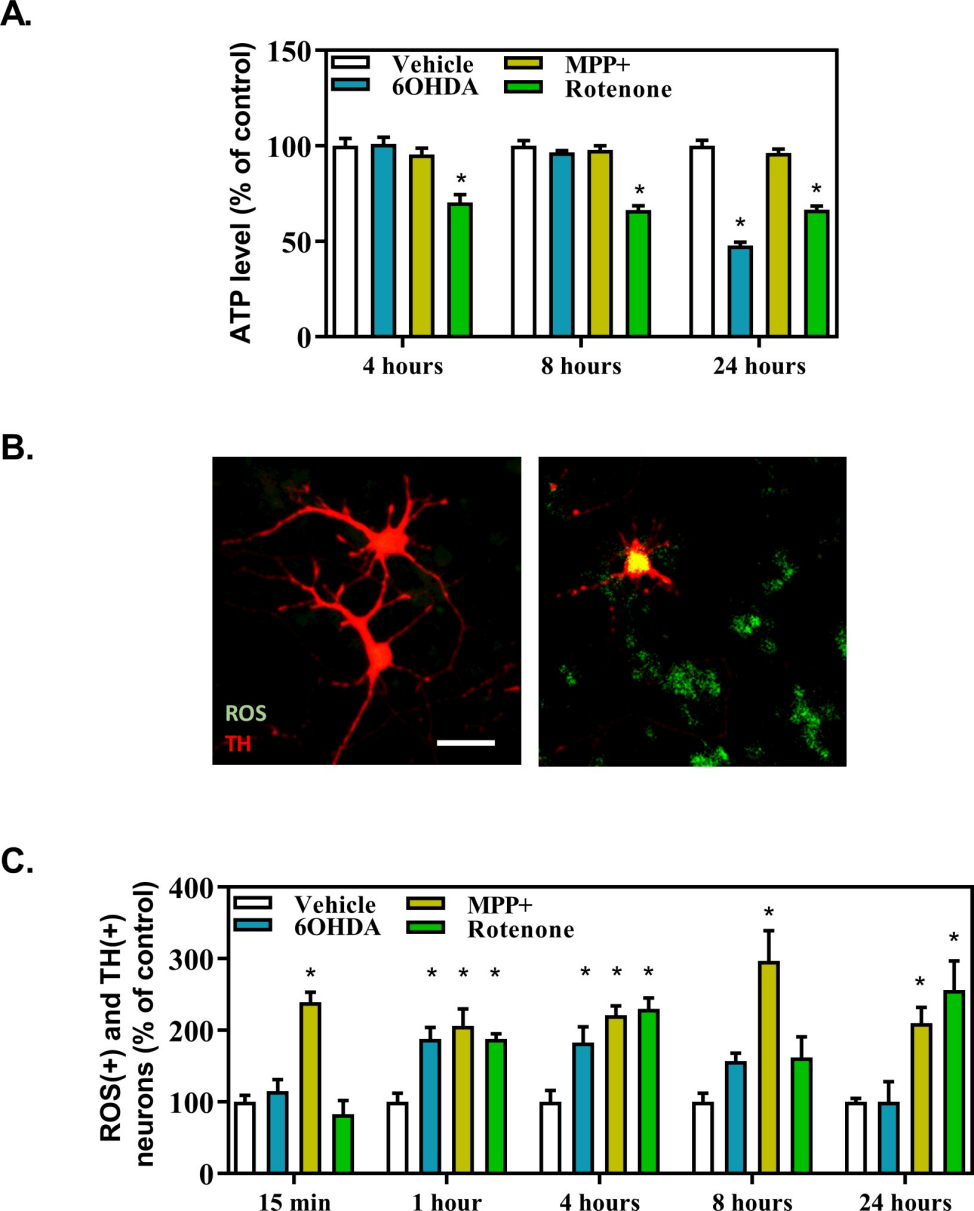
Mitochondrial pathology after DA-toxins application. Level of ATP normalized by cell viability (A) and oxidative stress (B-C) were determined at different time points. Representative pictures are showing TH expressing neurons with low level of ROS (left panel) or high level of ROS (right panel). A value of 1 corresponds to the mean intensity of luminescence in the vehicle condition (ATP) or to 5 +/- 1 neurons (ROS). All values were expressed as mean +/- SEM; n = 6/group; *, p<0,05 with One-way ANOVA followed by Dunnett’s test.

Among the three DA-toxins studied, only 6OHDA and rotenone induced a strong depletion in ATP production by mesencephalic cells (**Fig 5A**). Effects of rotenone appeared very early (after 4 hours of intoxication) and did not evolve with time. Depressive effects of 6OHDA appeared later (24 hours). It is worthy to note that there seemed to be a correlation between induction of necrosis and ATP depletion [19], including kinetics of appearance, ATP depletion being known to trigger necrosis, although MPP^+^ did not have a massive impact on ATP level.

ROS production was studied between 15 minutes and 24 hours after toxin application. All toxins were able to increase the number of TH-expressing neurons affected by an increase in ROS production (**Fig 5B and 5C**). Notably, ROS production was increased shortly after MPP^+^ application (15 min) and remained high and constant through time. 6OHDA and rotenone have induced a detectable production of ROS, which started after 1 hour of application and was transient.

### Effects of the DA-toxins on α-syn aggregation in TH-expressing neurons and possible release of LB-like inclusions

**Fig 6:**
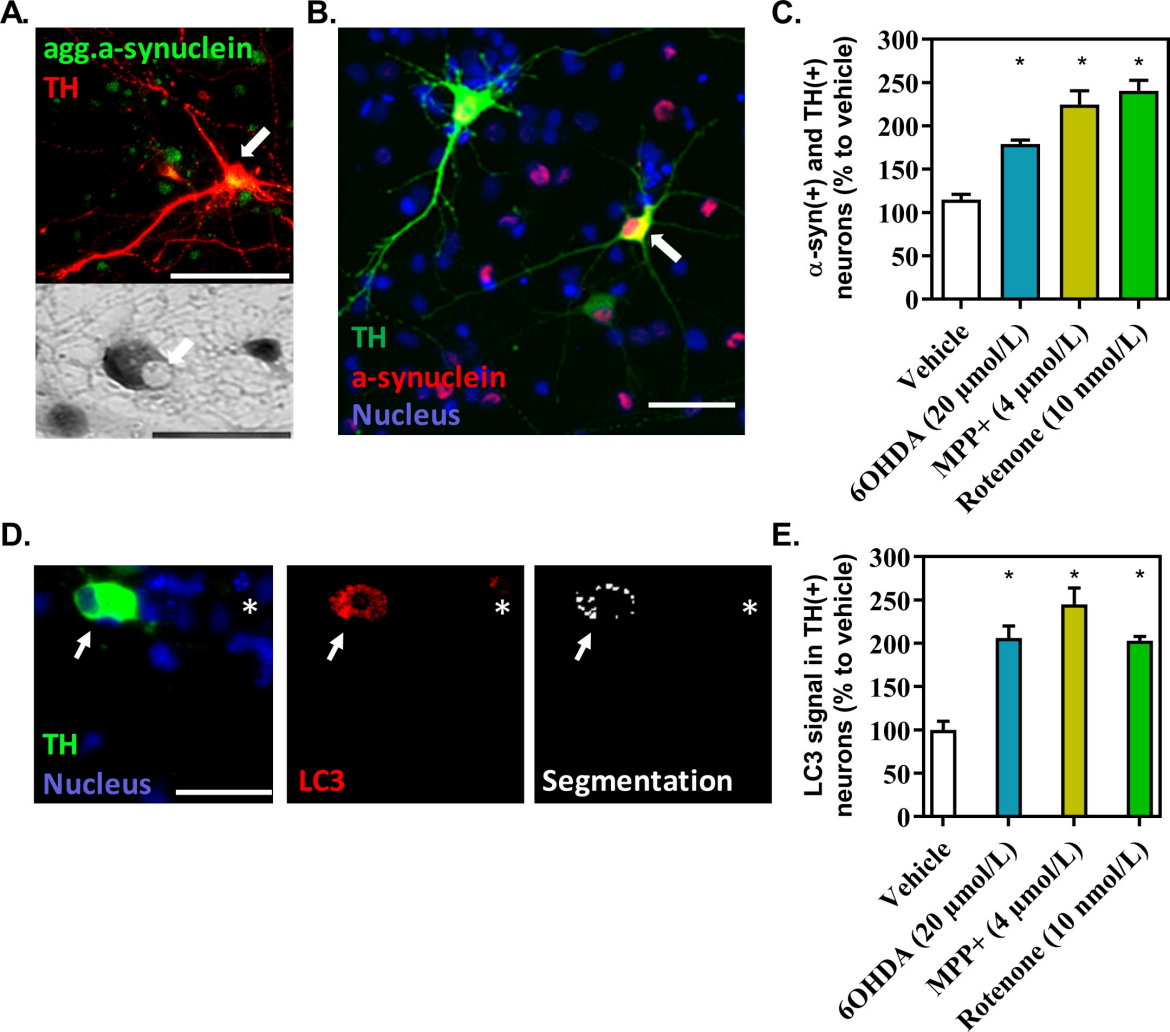
Alpha-synucleinopathy in dopaminergic neurons after exposure to 6OHDA, MPP^+^ or rotenone. **(A).** Upper panel: Representative picture of aggregated α-syn (in green) inside TH-expressing neurons (in red) (white arrows) after double-immunofluorescence microscopy, obtained with an antibody anti-aggregated α-syn (scale bar 100 μm). Lower panel: Representative picture of phase contrast microscopy reveals intracellular Lewy body-like structures (scale bar 100 μm). **(B, C).** Representative picture of α-syn, obtained with an antibody anti-α-syn and the area of α-syn staining overlapping with TH staining, corrected by the number of TH neurons, after an injury with 6OHDA (48 h), MPP+ (48 h) or rotenone (24 h). A value at 1 is equivalent to 7,1 +/- 0,38 neurons. **(D).** Representative pictures of LC3 staining (red) in TH expressing neurons (green) and the segmentation resulting from data processing (white). Arrow: TH neurons; star: non TH neuron showing LC3 staining. Data processing removed background signal to consider only dot structures in TH expressing neurons. **(E).** LC3 staining in dopaminergic neurons in presence of the toxins. A value at 1 is equivalent to 1.7 +/- 0,2 neurons. All values were expressed as mean +/- SEM; n = 6/group; *, p<0,05 with One-way ANOVA followed by Dunnett’s test.

Accumulation and aggregation of α-syn is a key hallmark of Parkinson’s disease. The three DA-toxins induced an apparent aggregation (as shown by the specific detection of the aggregated form of alpha-synuclein band by production of LB-like inclusions, **Fig 6A**) of α-syn. After 48 hours of application, 6OHDA (20 μmol/L) and MPP^+^ (4 μmol/L) significantly increased area of total alpha-synuclein staining in dopaminergic neurons showing an accumulation of α-syn (**Fig 6B**). Neuronal loss is rapid after 48 hours of a rotenone injury. For this reason, the accumulation of α-syn was investigated 24 hours after the application of rotenone. At this time point, a massive appearance of α-syn was observed at 10 nmol/L. In the three cases, an accumulation of α-syn were observed inside dopaminergic neurons.

The presence of α-syn aggregates increases the burden of protein clearance systems such as maroauthophagy [20]. Macroautophagy is a mechanism implying autophagosomes, which engulf misfolded proteins and injured cytosolic organelles such as mitochondria. During the progression of autophagy, cytosolic LC3-I is conjugated to phosphatidylethanolamine to form LC3-II. LC3-II is located in the inner and outer membranes of autophagosomes. Autolysosomes are then formed by fusing autophagosomes with lysosomes in order to degrade their content. We investigated the expression of LC3 in TH-expressing neurons after 24 hours of exposure to the DA-toxins by ICC, rather than WB, which is not cell specific. A specific program was applied during automatic data analysis to select only LC3 positive dot-like vesicles, which were considered as a surrogate marker for autolysosomes **(Fig 6D)**. We observed a significant increase of dot-like LC3 signal after the application of MPP^+^, 6OHDA and rotenone. We inferred that macroautophagy is stimulated in response to the intoxication and to the abnormal accumulation of α-syn.

## Discussion

For the first time, to the best of our knowledge, the mode of cell death induced by DA-toxins (usually used as an inducer of PD in animals) on primary culture of mesencephalic dopaminergic neurons was dissected and compared. As already shown in literature, we observed that all these toxins were able (at different levels of intensity) to induce aggregation of α-syn in dopaminergic neurons [21–23]. Under our conditions, we also observed that many α-syn aggregates were found outside the neurons in the culture. These aggregates looked like LB, known to mainly consist of aggregated α-syn.

**Fig 7:**
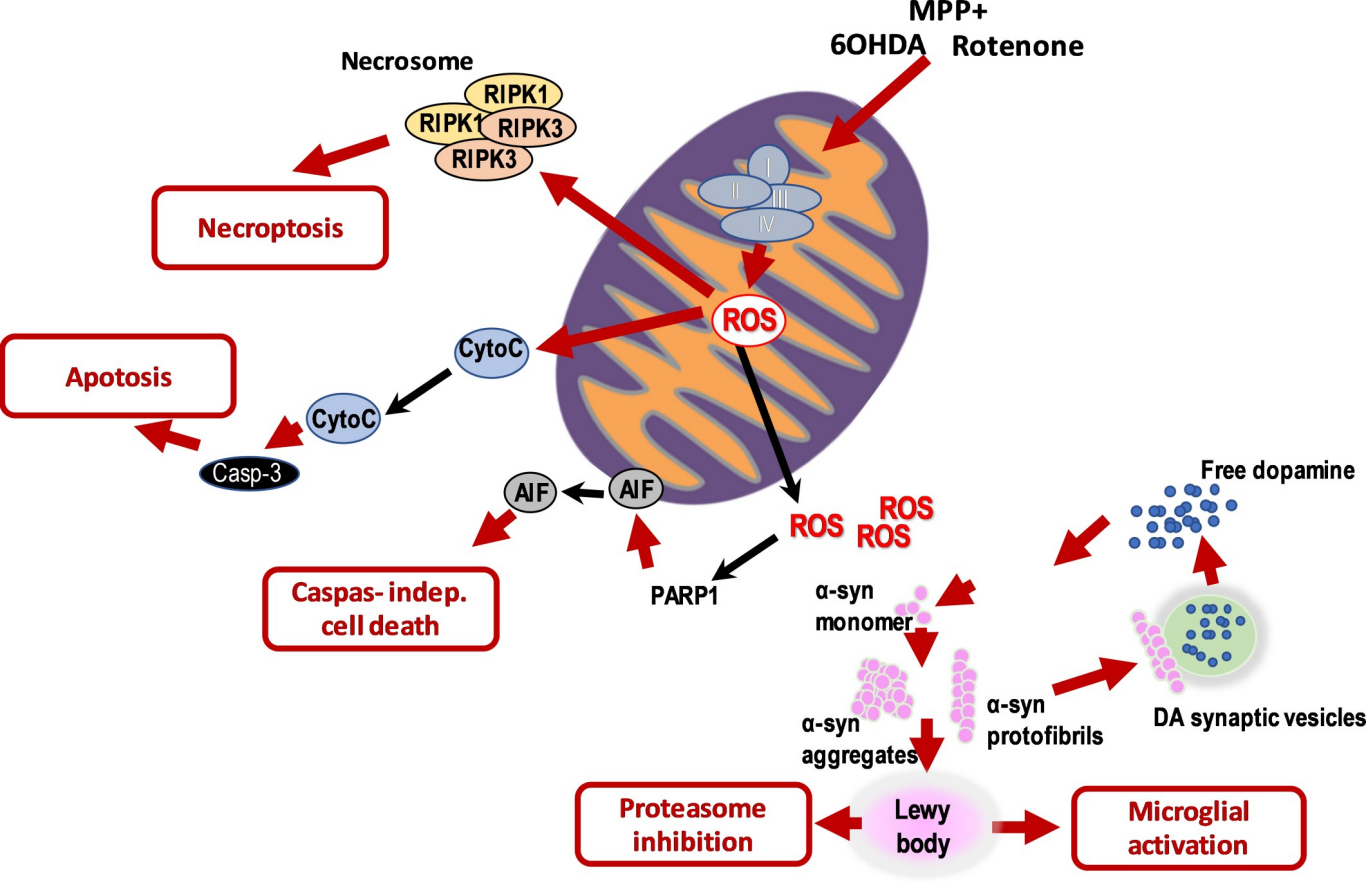
Schematic representation of the pathological events induced by the DA-toxins.

The three toxins used in this study were accepted to act on mitochondria and more specifically on mitochondrial complex 1, with different degrees of intensity (Fig 7). This does not exclude that they act on other cellular targets. However, these three cytopathic effects oriented the discussion of these results toward a link between induction of mitochondrial functioning impairment and α-syn involvement in dopaminergic neuron death: production of ROS, CytC release, and ATP depletion on the one hand, and extension of dopaminergic neuronal death on the other. We shall therefore concentrate the discussion on mitochondrial effects of the three DA-toxins.

6OHDA is known to inhibit both complexes I and IV in the mitochondrial respiratory chain. Here we showed that 6OHDA induced an early (4 h) oxidative stress associated with a large release of CytC, and activation of caspase 3 leading to apoptotic cell death within the 24 hours following its application. Whatever the dose of toxin used, we only detected apoptosis. These findings were in accordance with literature showing that 6OHDA massively increased ROS production in mitochondria and cytosol via auto-oxidation, within the first minutes following its application [24]. 6OHDA was imported faster in dopaminergic neurons thanks to DA transporters [25] and quickly auto-oxidized into the mitochondria. Twenty-four hours after 6OHDA application, intracellular and, unexpectedly, extracellular α-syn aggregates were observed in the culture, consistent with what had already been seen in 6OHDA animal models [26]. Endoplasmic reticulum stress could play a role in 6OHDA toxicity[26]. Finally, Giordano and colleagues [27] proved that 6OHDA shares none of the characteristics of an inhibitor of direct or indirect mitochondrial oxidative phosphorylation, which is in accordance with the oxidative stress we observed after 6OHDA application. Selegiline, indeed, protects neurons from 6OHDA injuries by reducing the production of ROS, upregulating superoxide dismutase and catalase, and suppressing non-enzymatic, iron-catalyzed auto-oxidation of DA [28,29] (see **Fig 7**). Altogether, these data are in favor of an early oxidative stress leading to neuronal death by apoptosis.

Rotenone is a widely used chemical inducer of PD-like symptoms in animals [30]; it is said to be an inhibitor of complex I of the mitochondrial respiratory chain [23]. Rotenone-induced PD replicates many features of sporadic PD including α-syn production and aggregation. We showed that rotenone exerted a massive toxic effect on TH-expressing neurons, dependent on dose and time of application. It induced an important necrosis of neurons, especially at the highest concentrations, whereas at low concentrations it only triggered apoptosis. It induced a decrease in ATP pools, and a large CytC release (after 8 hours of treatment). This is in accordance with what was previously shown by Pei and colleagues [31] on primary cortical neurons: rotenone, indeed, was proven to induce activation of the permeability transition pore, mitochondrial swelling, decrease in mitochondria-driven ATP levels, and induced caspase-3 independent cell death. In addition, we cannot exclude any necroptotic effect, as suggested by the increase of RIPK3, probably linked to the massive production of ROS as already shown in the literature [32].

We observed that treatment with rotenone (10 nmol/L) induced formation of α-syn inclusions, reminiscent of LB, in TH-expressing neurons. Only at low doses, large inclusions of α-syn were seen in their cytosol; at the highest doses (probably linked to occurrence of necrosis) α-syn aggregates were observed outside of TH-expressing neurons.

Rotenone (between 0.1 and 1 nmol/L) induced a large microtubule destabilization in TH-expressing neurons, that could disrupt vesicular transport along microtubule and cause the accumulation of DA vesicles in the soma[33]. This probably leads to the oxidative stress due to DA oxidation in the cytosol, and possibly to protein oxidation as shown by release of CytC (within 8 h) from mitochondria and triggering of the apoptotic cascade. An effect of rotenone on the mitochondrial complex 1 was suspected[34] although proved that in absence of complex I, dopaminergic neurons were still sensitive to rotenone. For the highest concentration (above 1 nmol/L), apoptosis was moderate and the cell death seemed to largely involve necrosis. ATP is indispensable for the formation of apoptosome complex and subsequent activation of the caspase cascade[35]. Rotenone quickly depleted (by 35%) ATP level in mesencephalic cells; this could explain the lack of activation of caspase 3. Kang and colleagues[36] showed indeed that rotenone at high doses (10 nmol/L) induced a dysregulation of apoptosis via the depletion of ATP that blocked the formation of an adequate apoptosome complex and then induced a caspase-independent necrotic phenomenon after mitochondrial release of CytC.

MPP^+^ is actively transported inside dopaminergic neurons via DA transporters; it would block complex I of the respiratory chain, although, here again, Choi and colleagues[34] showed that the complex I was not required for the toxicity of this toxin to be expressed. In fact, no apoptosis or oxidative stress occurred after MPP^+^ application. Contrary to rotenone, MPP^+^ did not induce ATP depletion within the first 24 hours. By contrast, 24 hours after application of MPP^+^, a non-negligible release of CytC was observed. Additionally, while probably inhibiting the respiratory chain as rotenone, MPP^+^ did not induce similar neurotoxicity. Some authors have suggested that it could act as a cation and thus impair the mitochondrial respiratory chain[27].

Moreover, the PARP-1 pathway was also involved in the degenerative process, and probably contributed to the overall mitochondrial defects. We also observed a large increase in mitochondrial AIF leakage. AIF is required for the maintenance of the mitochondrial respiratory complex I. Dysfunctions of mitochondria explained the massive production of ROS observed in presence of MPP^+^. As shown by the literature, necroptosis is a programmed form of cell death activated by extrinsic (e.g. cell membrane receptors such as FAS) or intrinsic stimulus, including the massive production of ROS[37]. Regulation of necroptosis signaling and cell death by reactive oxygen species). The increase of RIPK3 indicates that necroptosis was also involved in the MPP^+^ toxicity.

In parallel, aggregation of α-syn was observed inside the neurons, this accumulation was followed by a significant increase in LC3 (a marker of autophagy) after application of MPP^+^. This finding may support a potential role of autophagy at the early stage of MPP^+^ toxicity, to remove the protein aggregates[38].

To summarize, our results suggest that oxidative stress induced TH-expressing neuron death as well as production of aggregates of α-syn. These aggregates were localized inside and outside the neurons. Extracellular α-syn could activate the microglial cells. The uncontrolled activation of microglia may directly affect neurons by releasing various inflammatory mediators[39]. In turn, they could induce oxidative stress, generating new aggregates in unaffected neurons, contributing to the development of neurodegenerative processes. Alteration of mitochondrial electrical functions without major oxidative stress (such as that done by MPP^+^ acting as a cation and inducing depolarization) induced autophagy. Depolarization of mitochondria, PINK1 and parkin modulate transport of damaged mitochondria to the perinuclear region for autophagy[40]. This pathway metabolizes misfolded or aggregated proteins. Indeed, correct autophagy-lysosomal activities seem to be essential to prevent neurodegenerative diseases by removing damaged or dysfunctional proteins and organelles[27]. When the system is overloaded then necroptosis and cell death would occur.

In this paper, we unraveled in more detail the mode of action of three toxins (traditionally used *in vitro* and *in vivo* to mimic either cytopathological or clinical features of PD) on primary dopaminergic neurons in culture. We identified different processes of death induced by DA-toxins in dopaminergic neurons in primary culture. It seemed that any mitochondrial disturbances induced aggregation of α-syn protein. The intracellular aggregates contribute to the cell death. They could also be found outside of the cells as α-syn aggregates or LB-like structures which activate non-cell autonomous pathological processes, such neuroinflammation; α-syn would act in two ways on TH neuronal death, directly on mitochondria, when intracellular, and on microglia, via inflammatory cytokines, when extracellular. Such a vicious circle could explain the propagation of the TH neuronal death occurring in PD.

## Supporting information

S1 Table List of antibodies used in the study. (DOCX)

S1 Fig Early increase of CycC levels, 8 hours after ^2^MPP^+^ and rotenone application. (PDF)
