# Supplementary material for: Necrosis, apoptosis, necroptosis, three modes of action of dopaminergic neuron neurotoxins
Source: PLoS One. 2019 Apr 25;14(4):e0215277. doi: 10.1371/journal.pone.0215277 (PMC6483187; doi:10.1371/journal.pone.0215277)
Supplement: S1 Table — (DOCX) [file pone.0215277.s001.docx]

**SUPPORTING INFORMATION**

S1 Table. List of Ab used in the present study

| Antibody | Characteristics | Fixation | Dilution | Provider | Reference |
| --- | --- | --- | --- | --- | --- |
| Apoptosis inducing factor (AIF) | Polyclonal, rabbit | PFA | 1/50 | Santa cruz biotechnology | SC5586 |
| aggregated alpha-synuclein (α-syn) | Monoclonal, mouse | PFA | 1/200 | BioLegend | BLE847801 |
| alpha-synuclein (α-syn) | Polyclonal, rabbit | PFA | 1/200 | Cell signaling | 2642S |
| Caspase 3 | Polyclonal, rabbit | PFA | 1/500 | Sigma Aldrich | C8487 |
| Cytochrome C (CytC) | Polyclonal, rabbit | PFA | 1/100 | Abcam | ab90529 |
| LC3B | Polyclonal, rabbit | PFA | 1/25 | Cell signaling | 4108S |
| RIPK3 | Polyclonal, rabbit | PFA | 1/200 | Novus | NBP1-77299 |
| Tyrosine hydroxylase (TH) | Monoclonal, Mouse | PFA | 1/10 000 | Sigma Aldrich | T1299 |
