# Supplementary figures and images for: Necrosis, apoptosis, necroptosis, three modes of action of dopaminergic neuron neurotoxins
Source: PLoS One. 2019 Apr 25;14(4):e0215277. doi: 10.1371/journal.pone.0215277 (PMC6483187; doi:10.1371/journal.pone.0215277)

Supp Fig 1

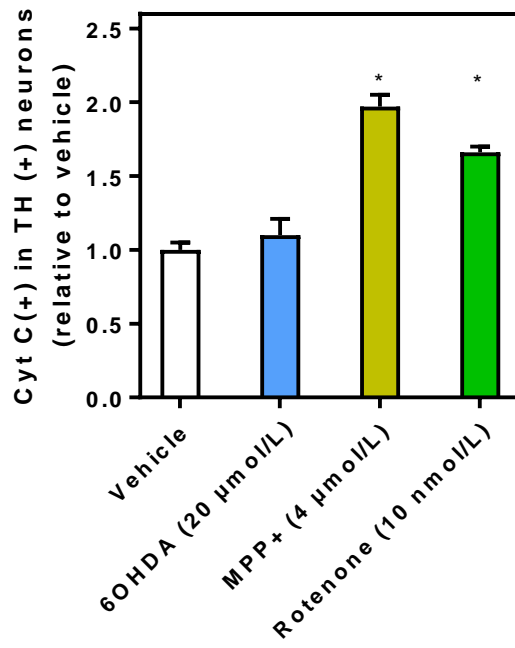

Supplement: S1 Fig — (PDF) [file pone.0215277.s002.pdf]
